# Sialic Acid Binding Properties of Soluble Coronavirus Spike (S1) Proteins: Differences between Infectious Bronchitis Virus and Transmissible Gastroenteritis Virus

**DOI:** 10.3390/v5081924

**Published:** 2013-07-26

**Authors:** Katarina Shahwan, Martina Hesse, Ann-Kathrin Mork, Georg Herrler, Christine Winter

**Affiliations:** Institute of Virology University of Veterinary Medicine Hannover, Germany; E-Mails: katkaren@t-online.de (K.S.), Ann-Kathrin.Mork@tiho-hannover.de (A.K.M.), martinahesse@hotmail.com (M.H.), Georg.Herrler@TiHo-Hannover.de (G.H.), Christine.Winter@TiHo-Hannover.de (C.W.)

**Keywords:** Coronavirus, spike protein, sialic acid receptor, TGEV, IBV

## Abstract

The spike proteins of a number of coronaviruses are able to bind to sialic acids present on the cell surface. The importance of this sialic acid binding ability during infection is, however, quite different. We compared the spike protein of transmissible gastroenteritis virus (TGEV) and the spike protein of infectious bronchitis virus (IBV). Whereas sialic acid is the only receptor determinant known so far for IBV, TGEV requires interaction with its receptor aminopeptidase N to initiate infection of cells. Binding tests with soluble spike proteins carrying an IgG Fc-tag revealed pronounced differences between these two viral proteins. Binding of the IBV spike protein to host cells was in all experiments sialic acid dependent, whereas the soluble TGEV spike showed binding to APN but had no detectable sialic acid binding activity. Our results underline the different ways in which binding to sialoglycoconjugates is mediated by coronavirus spike proteins.

## 1. Introduction

Transmissible gastroenteritis virus (TGEV) is a porcine alphacoronavirus and is a major pathogen for piglets unless they are protected by antibodies. The virus infects pigs via the oronasal route and infection of the intestinal epithelial cells results in disease with mortalities of up to 100 % in unprotected piglets. Binding of the virus to host cells is mediated via the viral surface glycoprotein S. The spike protein, a type I transmembrane protein, also has fusion activity. In the virus particle the S protein is present as a homotrimer. For TGEV, two binding properties of the S-protein have been described, the binding to the cellular receptor porcine aminopeptidase N and a sialic acid binding activity, which is important for the enteropathogenicity for the virus [1,2]. It is believed, that the sialic acid binding property helps the virus to bind to mucins on the surface of the epithelial cells of the gut. A naturally occurring variant virus of TGEV, the porcine respiratory coronavirus (PRCoV), lacks this sialic acid binding property due to a deletion in the N-terminal portion of the S1 subunit and is not enteropathogenic. Infection by this virus results in mild respiratory symptoms but elicits an effective immune response. Pigs that have been infected with PRCoV are protected against infection by TGEV. This “natural” vaccination has decreased the impact of TGEV infections in the porcine production enormously [3,4].

Sialic acid binding of the spike protein plays a role for many coronaviruses. In the genus *Betacoronavirus*, viruses like BCoV and HCoV-OC43 bind to 9-O acetylated sialic acid residues and have an additional membrane protein, the hemagglutinin-esterase, which has receptor-destroying activity. These viruses resemble influenza C viruses in their receptor usage and a specific protein receptor is not known [5]. During evolution, betacoronaviruses may have inherited a host galectin [6] and used it for their own attachment to target cells.

In the *Gammacoronavirus* genus, the infectious bronchitis virus is an important pathogen in the poultry industry. The spike protein of IBV binds to alpha 2,3 linked sialic acids. A protein receptor has not been identified so far. In contrast to BCoV and HCoV-OC43, the virus lacks a receptor-destroying enzyme. The presence of such an enzyme may be dispensable for IBV because of a relatively lower affinity to sialylated glycans [7]. 

In summary, there are coronaviruses with sialic acid binding activity in alpha-, beta- and gammacoronaviruses but they differ remarkably in their binding strategies. There are viruses like TGEV that require the sialic acid binding activity only to support intestinal infections and there are viruses like BCoV that are dependent on sialic acids as are influenza viruses. IBV may have an intermediate postion. In this study we compared the sialic acid binding properties of the spike proteins of TGEV and IBV using soluble spike proteins containing an IgG Fc-tag. 

## 2. Results

### 2.1. Cloning and expression of soluble spike proteins

**Figure 1:**
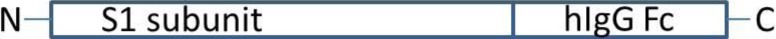
Schematic drawing of the soluble spike proteins. S1 subunits are fused c-terminally to the human IgG Fc portion.

The S1 portions of the genes of TGEV spike and IBV spike proteins were successfully cloned into the vector pCG1Fc where the open reading frames were in frame with the human IgG Fc tag. Transfection of BHK-21 cells with these plasmids resulted in the expression of soluble spike-Fc proteins which were secreted into the cell culture supernatant (Figure 1). After purification via protein A columns by FPLC, the proteins were used for binding tests. 

### 2.2. Soluble TGEV Spike protein binds to cells expressing APN

**Figure 2:**
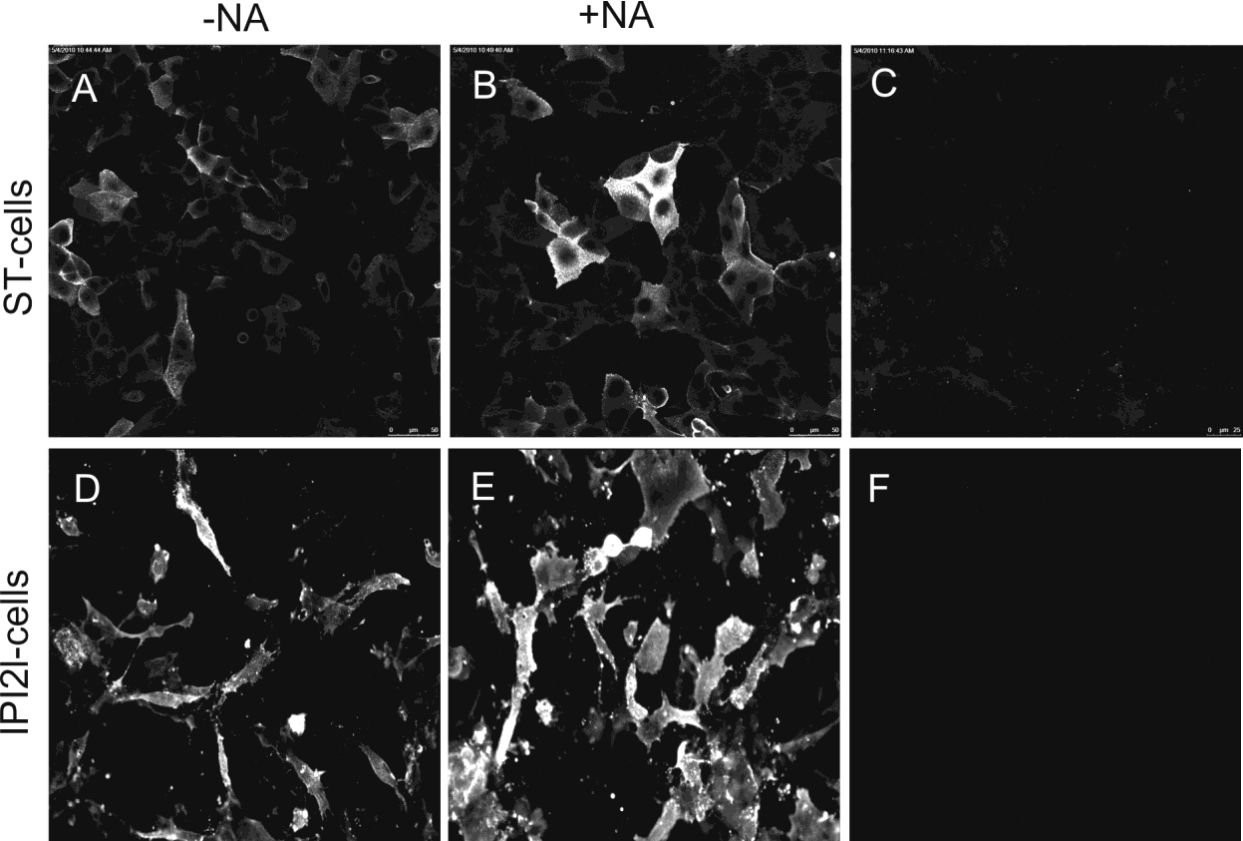
ST cells and IPI-21 cells, both of porcine origin were used for binding tests with soluble S transmissible gastroenteritis virus (TGEV)-S protein. Binding was evaluated immunofluorescence microscopy. Cells were either not treated (A, D) or pretreated with 100 mU of neuraminidase (B, E). Binding of soluble spikes was detectable on both neuraminidase treated and not treated cells. Control cells were incubated only with the secondary anti human FITC labeled antibody (C,F)

Soluble TGEV spike proteins were incubated with ST-cells and IPI-21 cells both expressing endogenous porcine aminopeptidase N, the receptor for TGEV. Binding of the soluble proteins was detected by immunofluorescence microscopy. Interestingly, a neuraminidase treatment of the cells, to remove sialic acids from the cell surface, resulted in an increased binding of the soluble S proteins (Figure 2). 

**Figure 3:**
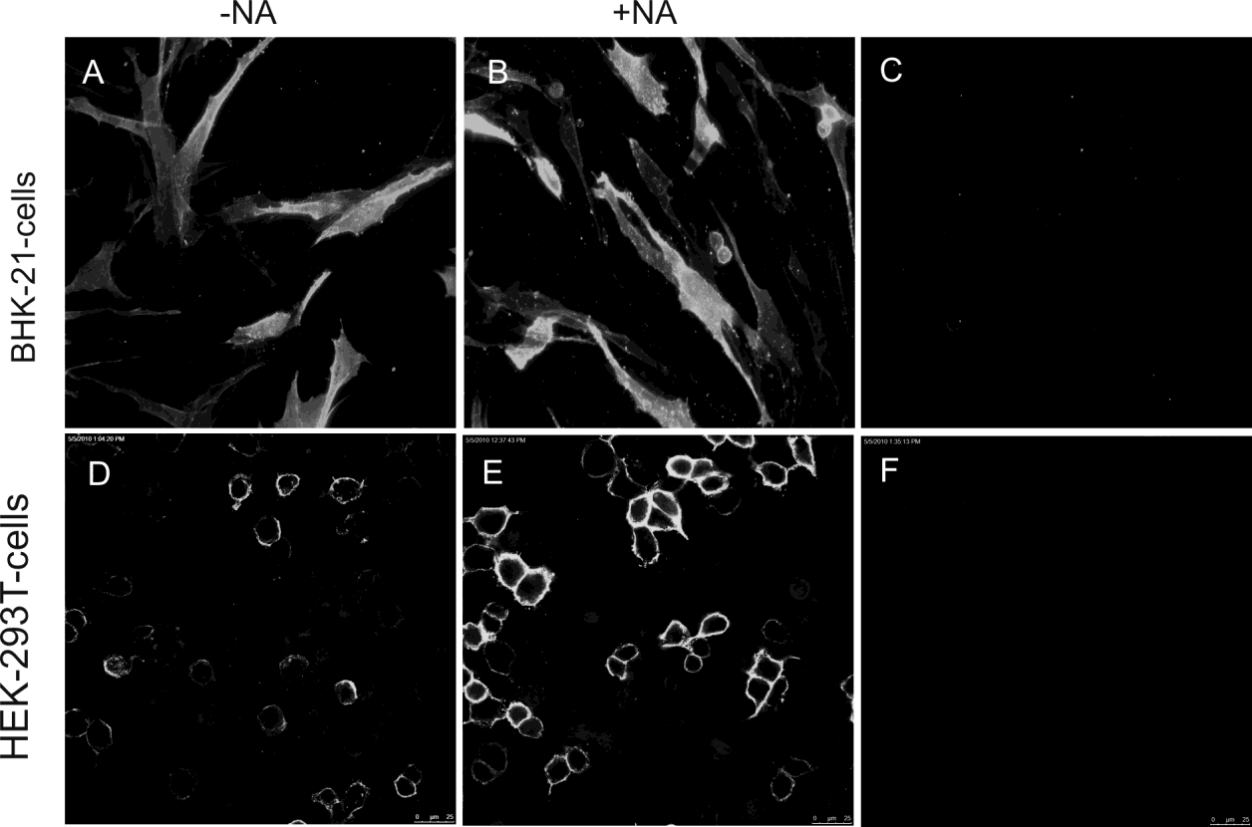
BHK-21 cells and HEK 293T cells were transfected with a plasmid for APN expression (A, B, D, E) and used for binding tests with soluble TGEV spike proteins. Binding was only detectable when porcine APN was expressed (A,B,D,E) and no binding was observed on cells transfected only with empty vector (C,F). A neuraminidase pre-treatment revealed an enhanced binding of the TGEV spike to the cells (B,E).

The TGEV spikes bound to cells of non-porcine origin (BHK or HEK-293T cells) only when the cells were transfected with plasmids for expression of the APN receptor. After desialylation of the cells by neuraminidase treatment, the binding was also enhanced (Figure 3). 

**Figure 4:**
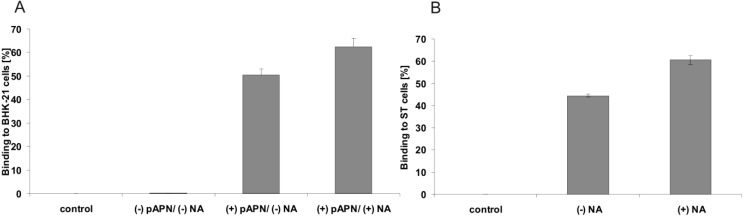
FACS analysis of soluble TGEV spikes bound to BHK-21 cells (A) and ST-cells (B). Binding was only detectable on BHK-21 cells expressing APN. A neuraminidase pre-treatment of the cells revealed an increased binding on both cell lines.

These results were confirmed by FACS analysis. Without neuraminidase treatment of the ST cells or BHK-21 cells expressing APN, the soluble spike proteins bound to approx. 50 % of the cells. This value was increased after removal of sialic acids from the cell surface to more than 60% of the cells (Figure 4).

### 2.3. Binding of soluble TGEV spike is not decreased after neuraminidase treatment of intestinal cells

**Figure 5:**
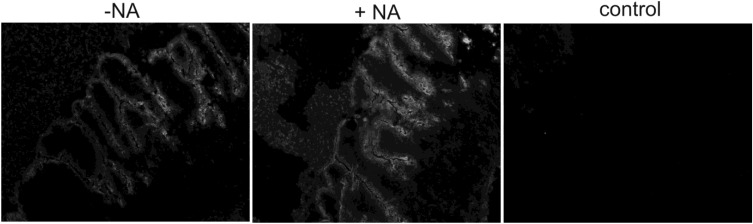
Binding test of soluble TGEV spike protein on cryosections of porcine jejunum: cryosections were either pretreated (+NA) or not treated (-NA) with 300 mU neuraminidase prior to binding of soluble spikes. The control cryosections were incubated only with the fluorescence dye (CY3) labeled secondary antibody. No decrease in the binding of TGEV spikes was observed after neuraminidase treatment.

As the sialic acid binding property of TGEV has been shown to support viral binding under unfavourable conditions, e.g., in the porcine intestine [8], we analyzed the binding properties of the soluble TGEV spike proteins on cryosections of porcine gut tissue. Binding of TGEV spikes to the brush border membrane was clearly detectable as shown in Figure 5. A pre-treatment of the cryosections with neuraminidase did not result in a reduced binding of the soluble spike protein to the cells indicating, that sialic acids play no role for the binding of the soluble TGEV spike to the intestinal epithelial cells. 

### 2.4. Soluble IBV Spike binds to chicken host cells in a sialic acid dependent manner

**Figure 6:**
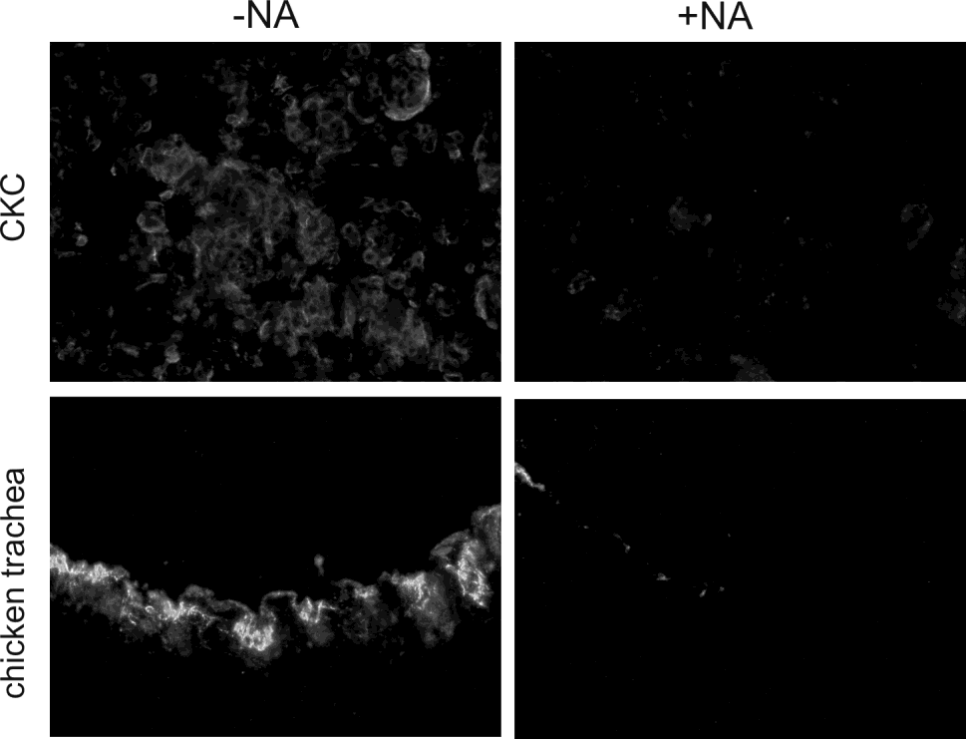
Sialic acid dependent binding of soluble IBV spike proteins to IBV-susceptible chicken cells: primary chicken embryo kidney cells (CKC) and cryosections of chicken trachea have been treated (+NA) or not treated (-NA) with 200 mU neuraminidase prior to the binding of soluble spike proteins. The binding is clearly reduced in the desialylated samples.

For comparison, we included the IBV spike protein in our analysis. We chose the cDNA coding for the S protein of the B1648 strain as this strain has no extended tropism to immortalized cell cultures like the Beaudette strain. We wanted to exclude any additional binding properties as, e.g., a binding to heparan sulphate, which has been reported for the Beaudette strain of IBV [9]. We observed a clear binding of the soluble IBV spike to IBV permissive cells like tracheal epithelial cells and primary chicken kidney cells (Figure 6). 

Binding of the IBV spike was detectable on kidney cells and on tracheal epithelial cells. A neuraminidase treatment of the cells reduced the binding of the soluble spikes (Figure 6). In contrast to the TGEV spike, the S protein of IBV binds to the respiratory epithelial cells in a sialic acid-dependent manner.

## 3. Experimental Section

### 3.1. Cells:

All cells were incubated at 37°C and 5% CO2. BHK-21 cells were maintained in Eagle minimum essential medium with 5% fetal calf serum, ST-cells and IPI-21 cells were maintained in Dulbecco´s essential medium and 10 % fetal calf serum. The latter cells received also L-glutamine, pyruvate and insulin.

Primary chicken embryo kidney cells were prepared as described previously [7] and maintained in Medium 199 and 5% fetal calf serum.

### 3.2. Cloning of soluble proteins:

Soluble spike proteins were generated by fusion of the S1 gene portion to the human IgG Fc gene and insertion into the vector pCG1. For TGEV, the cDNA of the strain Madrid (genbank accession number: M94101) was used and for IBV the cDNA of the B1648 strain (genbank accession number: X87238 ). 

### 3.3. Preparation of soluble proteins:

BHK-21 cells were transfected by calcium phosphate precipitation with expression plasmids containing the spike gene or with an empty vector as control. The supernatant of the transfected cells was collected at 24 and 48 hours post-transfection and pooled. The supernatant was either purified via FPLC by protein-A columns or concentrated by spinning through a 100 kilo Dalton filter device (Millipore). Both preparations worked well for binding tests.

### 3.4. Binding tests:

Cells and cryosections of tissue samples were incubated with the soluble spike proteins at a protein concentration of 2µg/µl or 50µl of concentrated supernatent for 1 hour at room temperature. The FITC or CY3-conjugated secondary antibodies (Sigma Aldrich) directed against the human Fc-tag, were incubated with cells or tissue slices also for 1 hour at room temperature. In the case of BHK-21 cells and HEK 293-T cells, cells were transfected 24 hours prior the binding test with a porcine aminopeptidase N gene containing plasmid to obtain receptor expressing cells. Control cells were transfected with empty vector. For pre-treatment with neuraminidase, the cells were incubated, if not otherwise stated, with 200 mU of neuraminidase from Clostridium perfringens type V (Sigma Aldrich). 

### 3.5. FACS analysis:

Single-cell suspensions were obtained by adding accutase to cell monolayers. Sialic acids were released with 200 mU neuraminidase. After three washing steps, the soluble spike protein was added in a concentration of 2µg/µl and cells were incubated for 1 hour at room temperature. After three washing steps, the cells were incubated with a FITC-labelled secondary antibody directed against the human Fc tag. FACS analysis was performed with a Beckman coulter FACS.

## 4. Conclusions

Binding to host cells is the first and a crucial step in a virus life cycle. For coronaviruses, binding is mediated by the viral spike protein. Within the spike protein there are two potential receptor binding domains (RBDs) both located in the S1 subunit. The RBDs been described to be responsible for binding to either a specific protein or to sialic acid [10]. The sialic acid binding site is located closer to the N-terminus Here we analyzed two different coronavirus spike proteins from an alpha- and a gammacoronavirus. The TGEV spike protein uses both RBDs, *i.e.*, binding to APN by the C-terminal RBD of S1 and binding to sialic acids via the N-terminal RBD of S1. The latter may help the virus to infect cells under unfavorable conditions [8]. We observed in this study, that the ability to bind to sialoglycoconjugates is not maintained when the protein is expressed in a soluble form which lacks a trimeric structure as it is present in the virion. In contrast, the binding property towards the receptor porcine APN is maintained. This is demonstrated by our binding tests with soluble spike proteins on cells expressing the receptor APN. Only if this protein is expressed, could the soluble proteins bind to the cells. Interestingly, a neuraminidase pretreatment of the cells enhanced the binding of TGEV-S. We conclude from this, that our soluble spike proteins have lost the ability to bind to sialic acids and that the neuraminidase treatment rendered the epitopes on cellular APN more accessible for interaction with the S protein. Crystal structures of TGEVand PrCoV RBDs complexed with porcine APN revealed an interaction of both CoV spike proteins with a glycan present in the APN region where the RBD binds. As the proteins used for crystallization experiments were generated using CHO-Lec cells, a group of CHO cells defective in some glycosylation pathways, it is not clear whether this interaction involves a terminal sialic acid, or whether it it is even enhanced if the glycan is desialylated, which would be an explanation of our results [11].

Soluble TGEV spike proteins carrying the trimerization domain of a leucine zipper protein have also been analyzed in this study but revealed in all cases a weaker binding compared to the Fc constructs (data not shown). In contrast to our results with TGEV, the sialic acid binding property of the gammacoronavirus IBV is effective when the spike protein is expressed in a soluble form even in the absence of a trimeric form. Binding of the IBV spike protein to host cells on cryosections of trachea and on primary chicken kidney cells was sialic acid-dependent. Our results are in contrast to the results of Verheije *et al.* [12] who reported no binding of a soluble B1648 S1 protein to sections of trachea and kidney. A weak binding to kidney cells was only observed if the sections had been treated with neuraminidase. An explanation for this contradictory result may be, that they used different soluble constructs where the eoctrodomain has been fused to a trimerization domain instead of a Fc domain in our case. It is not clear if a slightly changed conformation may interfere with the binding properties. As mentioned above, we observed with the TGEV spike a weaker binding of a construct with trimerization domain compared to the Fc constructs (not shown). Anyhow, our binding results with the B1648 spike resemble the tissue tropism of the B1648 virus. The virus infects primary chicken kidney cells as well as tracheal organ cultures [13]. So, a binding of the IBV spike to these susceptible cells is in line with the viral tropism. Our results may indicate that both coronaviral sialic acid binding sites are differently exposed by the S1 subunits. The conformation of the spike appears to play an important role. TGEV which needs the sialic acid binding property only to infect the gastrointestinal tract may show this feature only in the interplay of three functional S1 subunits. In contrast, the sialic acid binding activity of the IBV spike protein may be functional on each S1 monomer and therefore allow the virus to bind more efficiently to its receptor determinants. As a protein receptor is not known for IBV, the differences may be explained by the different importance of sialoglycans for the viruses to establish an infection. The effect of desialylation of cells on infection is more pronounced in the case of IBV than it is observed with TGEV. We conclude from our data, that the binding to sialic acids is more important for IBV than it is for TGEV. 
